# SMC1A knockdown induces growth suppression of human lung adenocarcinoma cells through G1/S cell cycle phase arrest and apoptosis pathways *in vitro*

**DOI:** 10.3892/ol.2013.1116

**Published:** 2013-01-08

**Authors:** YI-FAN ZHANG, RUI JIANG, JIN-DONG LI, XING-YI ZHANG, PENG ZHAO, MIAO HE, HOU-ZHONG ZHANG, LI-PING SUN, DONG-LEI SHI, GUANG-XIN ZHANG, MEI SUN

**Affiliations:** 1 Department of Thoracic Surgery, The Second Hospital of Jilin University, Changchun 130041;; 2 Department of Orthopedics, China-Japan Union Hospital of Jilin University, Changchun 130033;; 3 Departments of Anesthesia, The Second Hospital of Jilin University, Changchun 130041, P.R. China; 4 Pathology, The Second Hospital of Jilin University, Changchun 130041, P.R. China

**Keywords:** SMC1A, proliferation, shRNA, lung cancer

## Abstract

SMC1A (structural maintenance of chromosomes 1A), which encodes a structural subunit of the cohesin protein complex, is necessary for the process of sister chromatid cohesion during the cell cycle. Mutation and deregulation of SMC1A are highly relevant to diverse human diseases, including Cornelia de Lange syndrome and malignant carcinomas. In order to further investigate the role of SMC1A in the oncogenesis of lung cancer, SMC1A-specific short hairpin RNA (shRNA)-expressing lentivirus (Lv-shSMC1A) was constructed and used to infect A549 and H1299 cells. SMC1A mRNA and protein expression levels were downregulated in A549 and H1299 cells as demonstrated by real-time PCR and western blot assays. We found that SMC1A inhibition resulted in significantly impaired proliferation and colony formation as well as reduced invasiveness of tumor cells. Notably, Lv-shSMC1A-infected cancer cells exhibited a greater proportion of cells in the G0/G1 phase, but a lower proportion of S phase cells, compared to the parent or Lv-shCon infected cancer cells. Moreover, a greater proportion of sub-G1 apoptotic cells was observed in Lv-shSMC1A-infected cells. These results suggest that SMC1A is a novel proliferation regulator that promotes the growth of lung cancer cells, and that down-regulation of SMC1A expression induces growth suppression of A549 and H1299 cells via G1/S cell cycle phase arrest and apoptosis pathways. Therefore, SMC1A may serve as a new molecular target for lung cancer therapy.

## Introduction

Lung cancer is the most common malignancy and the leading cause of cancer-related mortality worldwide (1). Despite significant progress in surgical techniques and other conventional therapeutic modalities, such as chemotherapy and radiotherapy, most patients diagnosed with lung cancer succumb to the disease in a short period (2–4). Consequently, understanding the molecule mechanisms underlying the oncogenesis of lung cancer is crucially important for the development of more effective therapy of lung cancer (5–7).

The recent discovery of the cohesin complex in yeast has aided the further understanding of the molecular basis underlying genome instability, which has been recognized as a hallmark of human carcinomas (8). The cohesin complex, evolutionarily conserved from yeast to humans, comprises four subunits: a pair of SMC (structural maintenance of chromosomes) proteins, namely SMC1A and SMC3, and two non-SMC proteins, RAD21/SCC1 and STAG/SCC3/SA. SMC1A and SMC3 are composed of two coiled domains and interact with each other via their hinge domain to form an antiparallel heterodimer. Their head domains interact with RAD21, creating a ring-like structure (9). By trapping DNA within the ring-like structure, cohesin is associated with chromosomes, holding pairs of sister chromatids from the time of replication in S phase until their separation in anaphase to ensure faithful chromosome segregation during mitosis (10–12). It has been shown that the cohesin complex participates in a number of aspects of DNA repair, cell cycle, gene expression regulation and genomic imprinting, contributing to genome stability (13–15). Additionally, studies have demonstrated that the dysfunction of cohesin and cohesin regulatory genes makes them strong candidates for promoting genome instability and cancer development (16–18).

Previous studies have demonstrated that an increased risk of lung cancer is associated with deficiencies in DNA repair capacity, including in the DNA base excision repair genes XRCC1, PARP-1 and ERCC4 (19). The SMC1A gene maps to Xp11.22-p11.21 and consists of 25 exons, encoding a core component of the cohesin complex. In addition to the canonical role in sister chromotid cohesion, SMC1A is also known as a substrate of ATM/ATR protein kinases activated by specific DNA damage signaling, thereby playing a critical role in the regulation of gene expression and DNA repair (20–23). In recent years, the downregulated expression of SMC1A and other cohesin-related genes (NIPBL, SMC3, SCC3) caused by somatic mutations has been detected in colorectal cancers characterized by chromosome instability (CIN) (24). Moreover, researchers have reported that knockdown of SMC1A by RNA interference (RNAi) resulted in chromatid cohesion defects, mis-segregation and CIN *in vitro*(24,25). These findings imply that SMC1A may serve as a mutational target, whose disruption leads to the onset of CIN and cancer development. Apart from mutations, cohesin genes were found to be deregulated in diverse carcinomas. RAD21 and SMC3 were found to be overexpressed in breast and prostate cancer and colon carcinoma (26–28), while SMC1A and RAD21 were found to be downregulated in acute myeloid leukemia and oral squamous cancer (29,30). However, to date, the functional roles of SMC1A in human pulmonary carcinomas have not been demonstrated.

The RNAi technique, a powerful tool for carrying out loss-of-function assays, is a novel alternative to gene inhibition and provides a new approach for studying cancer gene therapy (31,32). Applications of RNAi for mammalian cells have emerged. In this study, we adopted a lentiviral vector-mediated RNAi system to achieve highly stable silencing of SMC1A. The safety of lentiviral vectors has been recognized in the scientific community (33).

In the present study, we constructed an SMC1A-specific small interfering RNA (siRNA)-lentiviral vector that is capable of effectively inhibiting the expression of the SMC1A gene in human lung adenocarcinoma A549 and H1299 cells and systemically investigated the impacts of SMC1A silencing on the growth and invasive ability of the cancer cells *in vitro*. Furthermore, we determined the effects of SMC1A knockdown on the cell cycle distribution and apoptosis of A549 and H1299 cells. As result, we found that SMC1A is a novel oncogeme, which modulates the proliferation and migration capabilities of lung cancer cells via G1/S phase cell cycle arrest and apoptosis.

## Materials and methods

### Cell culture

The human lung adenocarcinoma cell lines A549 and H1299 (Cell Bank of Chinese Academy of Sciences, Shanghai, China) and human embryonic kidney (HEK) 293T cell line (American Type Culture Collection, ATCC, Manassas, VA, USA) were maintained in DMEM (Hyclone, Logan, UT, USA) with 10% FBS (Hyclone) and penicillin/streptomycin at 37°C in humidified atmosphere of 5% CO_2_.

### Construction of SMC1A short hairpin (shRNA)-expressing lentivirus

To permit robust inducible RNAi-mediated SMC1A silencing, shRNA lentiviral vector was constructed. The RNAi was designed based on a 21-nt SMC1A (NM_006306)-targeting sequence (5′-TAGGAGGTTCTTCTGAGTACA-3′) of oligonucleotides and negative control sequence (5′-TTCTCCGAACGTGTCACGT-3′). The sequences were annealed and ligated into the *Nhe*I/*Pac*I- (NEB, Ipswich, MA, USA) linearized pFH1UGW vector (Shanghai Hollybio Co. Ltd., Shanghai, China). The lentiviral-based shRNA-expressing vectors were confirmed by DNA sequencing.

### Lentivirus infection

Recombinant lentiviral vectors and packaging vectors were cotransfected into 293T cells using Lipofectamine 2000 (Invitrogen, Carlsbad, CA, USA), according to the manufacturer’s instructions for the generation of recombinant lentiviruses [SMC1A shRNA (Lv-shSMC1A) and negative control shRNA (Lv-shCon)]. Supernatants containing lentiviruses expressing Lv-shSMC1A and Lv-shCon were harvested 72 h after transfection. Lentiviruses were purified using ultracentrifugation. A549 and H1299 cells were infected with the lentiviruses at a multiplicity of infection (MOI) of 30. Uninfected A549 and H1299 cells were used as controls.

### Quantitative real-time PCR

Quantitative real-time PCR was carried out using a previously described method (34,35). In brief, total RNA was extracted from A549 and H1299 cells 96 h after infection using the RNeasy Midi kit (Promega, Madison, WI, USA). cDNA was synthesized with SuperScriptII reverse transcriptase (Invitrogen). A mixture containing 1 *μ*g total RNA, 0.5 *μ*g oligo-dT primer (Shanghai Sangon, Shanghai, China) and nuclease-free water in a total volume of 15 *μ*l was heated at 70°C for 5 min and then cooled on ice for another 5 min. The mixture was supplemented with 2 *μ*l 10X buffer and 200 units Super-Script II reverse transcriptase to a final volume of 20 *μ*l, followed by incubation at 42°C for 60 min. Real-time quantitative PCR analysis was performed using SYBR-Green Master mix kit on DNA Engine Opticon™ system (MJ Research, Waltham, MA, USA). Each PCR mixture, containing 10 *μ*l 2X SYBR-Green Master mix (Takara, Dalian, China), 1 *μ*l sense and antisense primers (5 *μ*mol/*μ*l) and 1 *μ*l of cDNA (10 ng), was run for 45 cycles with denaturation at 95°C for 15 sec, annealing at 60°C for 30 sec and extension at 72°C for 30 sec in a total volume of 20 *μ*l. For relative quantification, 2-^ΔΔCt^ was calculated and used as an indication of the relative expression levels by subtracting CT values of the control gene from the CT values of SMC1A (36). The primer sequences for PCR amplification of the SMC1A gene were 5′-AAGTGAGGA GGAGGAGGAG-3′ and 5′-ACTTTCTTCAGGGTCTTG TTC-3′. β-actin was applied as an internal control. The primer sequences for β-actin were 5′-GTGGACATCCGCAAAGAC-3′ and 5′-AAAGGGTGTAACGCAACTA-3′.

### Western blot analysis

Western blotting was performed using our previously described method with modifications (34,35). In brief, A549 and H1299 cells were collected and lysed with precooled lysis buffer after 96 h of infection. Total protein was extracted from the cells and determined by the BCA method. Protein (20 *μ*g) was loaded onto a 10% SDS-PAGE gel. The gel was run at 30 mA for 2 h and transferred to polyvinylidene difluoride membrane (Millipore, Billerica, MA, USA). The resulting membrane was blocked in 5% non-fat dry milk blocking buffer and then probed with goat anti-SMC1A (1:1,000 dilution; Sigma, St. Louis, MO, USA; Cat. no. SAB4300451) and mouse anti-GAPDH (1:6,000; Santa Cruz Biotechnology, Inc., Sana Cruz, CA, USA) overnight at 4°C. The protein level of GAPDH was used as a control and detected by an anti-GAPDH antibody. The membrane was washed three times with Tris-buffered saline Tween-20 (TBST), followed by incubation for 2 h with anti-mouse IgG at a 1:5,000 dilution (Santa Cruz Biotechnology, Inc.). The membrane was developed using enhanced chemiluminescence (Amersham, UK). Bands on the developed films were quantified with an ImageQuant densitometric scanner (Molecular Dynamics, Sunny-Vale, CA, USA).

### Methylthiazol tetrazolium (MTT) assay

The MTT assay was performed using a previously described method (33). Briefly, exponentially growing cells were inoculated into 96-well plates with 2×10^3^ A549 cells or 6×10^4^ H1299 cells per well. After incubation for 24, 48, 72, 96 and 120 h, 10 *μ*l sterile MTT (5 mg/ml) was added into each well. Following incubation at 37°C for 4 h, the reaction was stopped by adding 100 *μ*l dimethyl sulfoxide. The formazan production was detected by measurement of the spectrometric absorbance at 595 nm. The values obtained are proportional to the amount of viable cells.

### Colony formation assay

The colony formation assay was performed using a previously described method (34). In brief, A549 and H1299 cells infected with Lv-shSMC1A or Lv-shCon and uninfected cells (Con) were seeded in six-well plates (2×10^2^ cells/well of A549, 5×10^4^ cells/well of H1299) and cultured at 37°C with 5% CO_2_ for 8 days. The cell colonies were washed twice with PBS, fixed in 4% paraformaldehyde for 15 min and stained with Giemsa for 30 min. Individual colonies with >50 cells were counted under a fluorescence microscope.

### Cell migration assay

The cell migration assay was performed using our previously described method (34). In brief, A549 and H1299 cells infected with Lv-shSMC1A or Lv-shCon for 96 h and uninfected cells (Con) were harvested and their ability to migrate *in vitro* was determined using a Transwell chamber (Corning, NY, USA). Cells were seeded into the upper chamber (3.0×10^4^ cells/well of A549, 8.0×10^4^ cells/well of H1299) in 100 *μ*l serum-free medium. Medium (1 ml) containing 20% FBS was added to the lower chamber as a chemo-attractant. After incubation for 24 h at 37°C in 5% CO_2_, cells that invaded to the lower surface of the filter were fixed in 4% paraformaldehyde and stained with crystal purple. Cell numbers were counted in five random fields (×100) per filter and detected by the spectrometric absorbance at 570 nm.

### Fluorescence-activated cell sorting (FACS) analysis

FACS flow cytometry analysis of cell cycle and apoptosis was performed using our previously described method (34). In brief, A549 and H1299 cells were seeded in six-well plates (A540, 1.5×10^6^ cells/well; H1299, 2×10^6^ cells/well). After 48 h, cells were collected, washed with PBS and fixed with 75% cold ethanol. The cells were then incubated for >24 h at 4°C. After washing the cells with PBS, propidium iodide (PI) was added to the cell suspension and the analysis of cell cycle distribution was performed by FACScan (Becton-Dickinson, Franklin Lakes, NJ, USA).

### Statistical analysis

Data are expressed as mean ± SD. Student’s t-test was performed to evaluate inter-group differences. P<0.05 was considered to indicate a statistically significant result. All statistical analyses were performed with SPSS 10.0 software (SPSS, Inc., Chicago, IL, USA).

## Results

### Efficacy of lentivirus-mediated RNAi targeting of SMC1A

**Figure 1:**
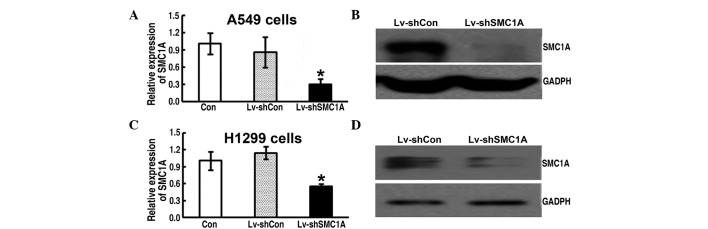
SMC1A mRNA and protein levels in human lung carcinoma A549 and H1299 cells were markedly downregulated in Lv-shSMC1A-infected cells, as evidenced by (A and C) real-time PCR and (B and D) western blot analysis. ^*^P<0.01 versus Con or Lv-shCon. Con, control; SMC1A, structural maintenance of chromosomes 1A.

To determine the silencing effect of lentivirus-mediated SMC1A RNAi on SMC1A expression in A549 and H1299 cells, real-time PCR and western blot analysis were performed after 72 h of infection. The expression level of SMC1A mRNA of the Lv-shSMC1A-infected cells was significantly lower than that of the parent (Con) and Lv-shCon-infected cells (Fig. 1A and C). Moreover, the western blot assay further showed that SMC1A protein levels were significantly decreased in Lv-shSMC1A-infected cells compared with those of Lv-shCon-infected cells (Fig. 1B and D). Therefore, this indicates the high efficacy of lentivirus-mediated SMC1A shRNA on SMC1A expression in lung cancer cells.

### Impact of downregulation of SMC1A expression on cell growth in vitro

**Figure 2:**
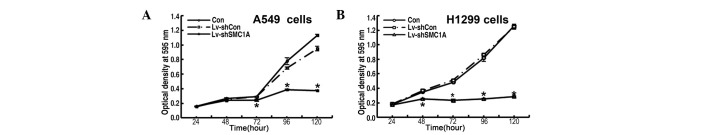
Proliferation of (A) A549 and (B) H1299 cells was inhibited following Lv-shSMC1A infection, as determined by MTT assay. ^*^P<0.01 versus Con or Lv-shCon. Con, control; SMC1A, structural maintenance of chromosomes 1A; MTT, methylthiazol tetrazolium.

**Figure 3:**
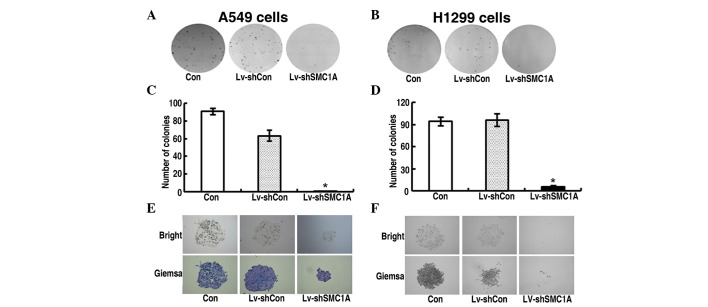
Growth of A549 and H1299 cells was inhibited following Lv-shSMC1A infection, as determined by colony formation assay. (A and B) Images of colonies. (C and D) Statistical analysis of the number of colonies. (E and F) Images of colonies recorded under microscope. ^*^P<0.01 versus Con or Lv-shCon. Con, control; SMC1A, structural maintenance of chromosomes 1A.

To explore the functional role of SMC1A in the proliferation of lung cancer cells, the growth dynamics of parent or Lv-shCon and Lv-shSMC1A-infected A549 and H1299 cells was determined by MTT and colony formation assays, respectively. The MTT assay showed that, during the 120-h incubation period, the growth of Lv-shCon-infected cells did not differ from that of the uninfected parent cells and showed strong proliferation, whereas the growth of Lv-shSMC1A-infected cells was markedly slower than that of the parent or Lv-shCon-infected cells at 48, 72, 96 and 120 h (Fig. 2). Quantitative analysis of colonies revealed that after incubation for 8 days, the number of colonies of Lv-shSMC1A-infected cells was lower than that of the parent and Lv-shCon-infected cells (P<0.01) (Fig. 3C and D). Therefore, the low viability and colony-forming efficiency of Lv-shSMC1A-infected A549 and H1299 cells demonstrated that downregulation of SMC1A expression inhibits the growth of lung cancer cells *in vitro*.

### Impact of downregulation of SMC1A expression on cell invasion

**Figure 4:**
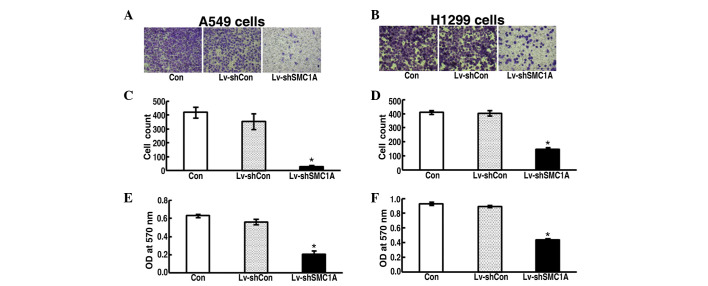
Lv-shSMC1A infection reduced the invasion of A549 and H1299 cells as determined by Transwell chamber invasion assay. (A and B) Images of migrated cells. (C and D) Number of migrated cells. (C and E) Quantitative analysis of migrated cells at 570 nm optical density. ^*^P<0.01 versus Con or Lv-shCon. Con, control; SMC1A, structural maintenance of chromosomes 1A; OD, optical density.

To determine the role of SMC1A in lung cancer invasion, we tested the invasive ability of parent and Lv-shCon- or Lv-shSMC1A-infected A549 and H1299 cells using the Transwell chamber assay 96 h after infection. As shown in Fig. 4, the invasive ability of Lv-shCon-infected cells did not significantly differ from that of the parent cells and showed strong invasiveness. However, the invasive ability of Lv-shSMC1A-infected cells was markedly lower than that of the parent and Lv-shCon-infected cells (Fig. 4C–F). Therefore, this indicates that the downregulation of SMC1A expression mitigates the invasion of lung cancer cells *in vitro*.

### Impact of downregulated SMC1A expression on cell cycle distribution in vitro

**Figure 5:**
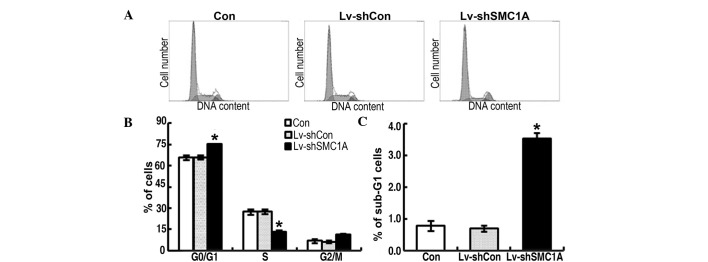
Fluorescence-activated cell sorting (FACS) analysis of A549 cells demonstrated that Lv-shSMC1A infection induced cell cycle arrest at the G1/S boundary and triggered apoptosis. (A) Histograms of FACS analysis. (B) Cell cycle distribution. (C) Percentage of sub-G1 phase cells. ^*^P<0.01 versus Con or Lv-shCon. Con, control; SMC1A, structural maintenance of chromosomes 1A.

**Figure 6:**
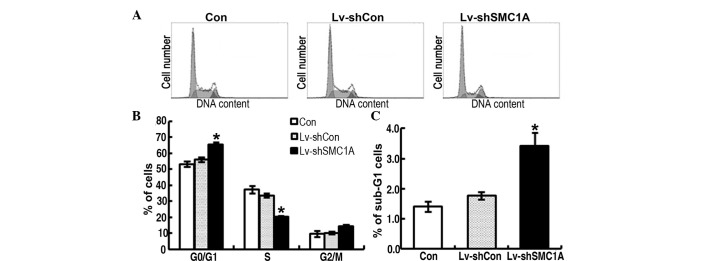
Fluorescence-activated cell sorting (FACS) analysis of H1299 cells. (A) Histograms of FACS analysis. (B) Cell cycle distribution. (C) Percentage of sub-G1 phase cells. ^*^P<0.05 versus Con or Lv-shCon. Con, control; SMC1A, structural maintenance of chromosomes 1A.

To explore the potential mechanism underlying the action of SMC1A in the growth of A549 and H1299 cells, the cell cycling patterns of parent, Lv-shConand Lv-shSMC1A-infected cancer cells were determined by FACS flow cytometric analysis 96 h after infection. As shown in Fig. 5B and Fig. 6B, there was no evident difference in the frequency at G2 stage of each group of cells. The frequency of Lv-shCon-infected cells at G1 and S stage did not significantly differ from its parent cells. At G1 stage, the frequency of Lv-shSMC1A-infected cells was significantly higher than that of its parent or Lv-shCon-infected cells. By contrast, at S stage, the frequency of Lv-shSMC1A-infected cells was lower than that of the controls (P<0.01) (Figs. 5B and 6B). These results suggest that the downregulation of SMC1A expression resulted in cell cycle arrest at the G1/S transition in A549 and H1299 cells, which contributed to the inhibition of SMC1A cell growth.

### Impact of downregulated SMC1A expression on apoptosis in vitro

To detect the apoptosis, sub-G1 phase cells were measured. Such cells are usually considered to be the result of apoptotic DNA fragmentation: during apoptosis, the DNA is degraded by cellular endonucleases. Therefore, nuclei of apoptotic cells contain less DNA than nuclei of healthy G0/G1 cells, resulting in a sub-G1 peak in the fluorescent histogram that may be used to determine the relative amount of apoptotic cells (37,38). As shown in Figs. 5C and 6C, there was no marked difference in the cell population at sub-G1 phase between parent and Lv-shCon-infected cells, whereas Lv-shSMC1A-infected cells exhibited a significantly higher proportion in sub-G1 phase than that of parent or Lv-shCon-infected cells. This suggest that the downregulation of SMC1A expression may trigger apoptosis in lung cancer cells, contributing to the suppression of SMC1A cell growth.

## Discussion

Lung cancer is well established as a highly heterogeneous disease, with a multitude of cellular components and patterns of gene expression that affect tumor development (39). An in-depth understanding of the molecular mechanisms underlying cancer proliferation is critical for the development of optimal therapeutic modalities. Moreover, there is evidence to suggest that therapeutic drugs specifically targeting tumor-related molecules are expected to be highly specific to malignant cells and have minimal adverse reactions due to their actions through well-defined mechanisms. Cohesin is emerging as the master regulator of genome stability and its related genes have been found to be highly relevant to diverse human malignancies. In the present study, we determined the expression levels of SMC1A expression in lung adenocarcinoma A549 and H1299 cell lines using quantitative real-time PCR assay and western blot analysis, and observed clear expression of SMC1A in lung cancer cells. Consequently, this led to a hypothesis that, as an indispensible subunit of the cohesin complex, SMC1A may play a functional role in the biological behavior of lung cancer.

We adopted a lentiviral vector-mediated RNAi system to further determine the roles of SMC1A in the growth and invasive ability of lung cancer cells. Using a constructed lentivirus expressing SMC1A-specific shRNA, we infected A549 and H1299 cells to silence endogenous SMC1A and investigated the impact of SMC1A knockdown on the lung cancer development *in vitro*. We found that downregulation of SMC1A expression greatly impaired the proliferation and colony-forming ability of A549 and H1299 cells. Furthermore, our study also showed that SMC1A knockdown may greatly reduce the migration capacity of the lung cancer cecolls, as evidenced by the Transwell chamber invasion assay. Notably, we observed that SMC1A knockdown caused cell cycle arrest at the G1/S transition of A549 and H1299 cells, as evidenced by the accumulation of G1 phase cells and decrease in S phase. In addition, SMC1A silencing induced apoptosis, as characterized by the prominent presence of sub-G1 apoptotic cancer cells. Collectively, these findings are the first report that SMC1A is a novel regulator of proliferation in lung cancer.

The hallmarks of cancer involve several critical biological capabilities acquired during cell proliferation and the invasion-metastasis cascade of malignant tumors. Genome instability has been found to foster these multiple hallmarks and generates the genetic diversity that expedites their acquisition (40). Recently, cohesion defects are emerging as critical factors of genome instability that involve defects in DNA repair, cell cycle checkpoints and epigenetic processes (41). Studies have revealed that, apart from its role in sister chromatid cohesion, cohesin is also key in various aspects of DNA damage response, cell cycle and gene expression regulation (13–15). SMC1A, an indispensible component of the versatile cohesin complex, is implicated as an important molecular target in malignancies. Our observation found that SMC1A facilitates important regulatory roles in lung cancer cell proliferation and invasiveness. There is evidence to suggest that several factors are implicated in the genesis of lung cancer, including new fusion genes, new gene expression, changing expression of p53, growth factors, cytokines and chemokine receptors and STAT3 (signal transducer and activator of transcription 3) (39,42–45). However, to date, the issue of whether and how SMC1A interacts with other regulators is poorly understood, and further investigation is warranted to elucidate the detailed mechanisms underlying the action of SMC1A.

In conclusion, our findings strongly suggest the significance of the cohesin gene SMC1A in modulating the growth and invasiveness of lung cancer and indicate that downregulation of SMC1A expression induces growth suppression of human pulmonary adenocarcinoma A549 and H1299 cells via G1/S phase cell cycle arrest and apoptosis pathways. Hence, this study extends our knowledge of the oncogenesis of lung cancer, and indicates that SMC1A may serve as a new molecular target.
